# *Anemonastrum tenuicaule* and *A. antucense* (Ranunculaceae), new combinations for a New Zealand endemic species and its South American relative

**DOI:** 10.3897/phytokeys.99.26489

**Published:** 2018-05-30

**Authors:** Sergei L. Mosyakin, Peter J. de Lange

**Affiliations:** 1 M.G. Kholodny Institute of Botany, National Academy of Sciences of Ukraine, 2 Tereshchenkivska Street, Kyiv (Kiev), 01004, Ukraine; 2 Environment and Animal Sciences, Unitec Institute of Technology, Private Bag 92025, Victoria Street West, Auckland 1142, New Zealand

**Keywords:** New Zealand, South America, Ranunculaceae, *Anemonastrum*, *Anemone*, Anemoneae, new combinations, typification, biogeography

## Abstract

A rational taxonomic circumscription of genera in tribe Anemoneae (Ranunculaceae) is briefly discussed. It is concluded that, in view of the morphological diversity of the group and recent molecular phylogenetic findings, a moderately narrow approach to the re-circumscription of genera earlier included in *Anemone* sensu lato is preferable, in particular, with the recognition of the lineage with the base chromosome number *x* = 7 (Anemone subgen. Anemonidium) as two genera, *Hepatica* sensu stricto and *Anemonastrum* in an expanded circumscription (including *Anemonidium*, *Arsenjevia*, *Jurtsevia*, and *Tamuria*). Following these conclusions, new nomenclatural combinations are proposed for two related species endemic to New Zealand and South America, respectively: *Anemonastrum tenuicaule* (= *Anemone tenuicaulis*, *Ranunculus tenuicaulis*) and *Anemonastrum antucense* (= *Anemone antucensis*). Information on typification is updated: the lectotype of *Anemone antucensis* is the specimen from P and not a specimen from G, and the lectotype of *Ranunculus tenuicaulis* is a specimen from AK. Biogeographic scenarios already proposed to explain the relationship of these two species and some other South America – New Zealand distribution patterns are discussed. It is concluded that the long-distance dispersal scenario fits best the available data for *Anemonastrum*. Two host-specific and geographically restricted species of *Urosystis* parasitizing *A. tenuicaule* and *A. antucense* are briefly discussed.

## Introduction

Recent molecular phylogenetic results obtained for taxa of the tribe Anemoneae (Ranunculaceae) and, in particular, *Anemone* L. sensu lato and *Clematis* L. (see Hoot et al. 1994, 2012; Hoot 1995; Ehrendorfer 1995; Ehrendorfer and Samuel 2000, 2001; Schuettpelz and Hoot 2000, 2001; Schuettpelz et al. 2002; Meyer et al. 2010; Pfosser et al. 2011; Xie et al. 2011; Cossard et al. 2016; Lehtonen et al. 2016; Elliott 2016; Jiang et al. 2017), stimulated the long-standing discussion on a rational taxonomic circumscription of genera in that group. In particular, Mosyakin (2016) argued that the very broad taxonomic circumscription of *Anemone*, as outlined by Hoot et al. (2012) [including *Hepatica* Mill., *Pulsatilla* Mill., *Knowltonia* Salisb., *Barneoudia* Gay, *Oreithales* Schtdl., and many other generic segregates], is morphologically poorly justified. Moreover, if *Clematis* is indeed confirmed as phylogenetically rooted in *Anemone* sensu lato, as suggested by Lehtonen et al. (2016) and in some earlier publications (see discussion in Wang et al. 2009; Pfosser et al. 2011; Cossard et al. 2016), then the taxonomic recognition of *Anemone* (as outlined by Hoot et al. 2012) will be also unnatural from the phylogenetic viewpoint.

The new molecular phylogenetic results reported by Jiang et al. (2017) indicated non-monophyly of *Anemone* s.l. (in the wide circumscription accepted by Hoot et al. 2012), as revealed by plastid datasets. At least one of their tree topologies (based on the combined nrITS + *atpB*-*rbcL* datasets, the same markers as those used by Hoot et al. 2012), however, suggested the sister position of the clades of *Anemone* (incl. *Hepatica* etc.) and *Clematis* + *Anemoclema* (Franch.) W.T. Wang. These findings partly contradict but mostly confirm the results of Lehtonen et al. (2016), who reported that *Clematis* (with *Anemoclema* as the sister genus; see also Zhang et al. 2014) is phylogenetically rooted in *Anemone* sensu lato. However, there are some evident gaps in the sampling of taxa used by Jiang et al. (2017) in their analysis: in particular, no taxa of Anemone sect. Anemone and early-branching taxa of Anemone sect. Pulsatilloides DC. (sensu Hoot et al. 2012) were included, which may have resulted in different and distorted tree topologies. Further molecular phylogenetic studies involving all major subclades of Anemoneae are needed to clarify the position of *Clematis* in relation to taxa of *Anemone* sensu lato.

Jiang et al. (2017: 13) also provided “Recommendations for reclassification of tribe Anemoneae”, in which they stated that the “subgenus Anemoniudium [sic! *Anemonidium* – S.M. & P.dL.] (Spach) Juz. needs to be separated as an independent genus, *Hepatica*. In the new genus *Hepetica* [sic! *Hepatica* – S.M. & P.dL.], four sections were recognized, *Hepatica* Spreng., *Anemonidium* Spach, *Keiska* [sic! *Keiskea* – S.M. & P.dL.] Tamura, and *Omalocarpus* DC.”. However, if we accept that recommendation to expand the generic limits of *Hepatica* so dramatically, it will be highly disruptive for nomenclature because numerous new nomenclatural combinations will be required, resulting from transfers of many taxa of *Anemone* (sections *Keiskea* Tamura, *Anemonidium* Spach, and *Omalocarpus* DC. as accepted in Hoot et al. 2012) to the newly circumscribed *Hepatica*.

Other options of phylogenetically non-controversial and taxonomically rational re-circumscription of genera in the group of *Anemone* sensu Hoot et al. (2012) were recently discussed by Mosyakin (2016) who, in particular, advocated the recognition of the lineage with the base chromosome number *x* = 7 (Anemone subgen. Anemonidium sensu Hoot et al. 2012) as comprising two genera, *Hepatica* in its traditional circumscription and *Anemonastrum* Holub in an expanded circumscription, including *Anemonidium* (Spach) Holub, *Arsenjevia* Starod., *Jurtsevia* Á. Löve & D. Löve, and *Tamuria* Starod. The clade of “*Anemone*” with *x* = 7 and its two main subclades corresponding to the genera *Hepatica* and *Anemonastrum* in the circumscriptions proposed above were consistently and reliably revealed in all recent phylogenetic analyses (e.g., Pfosser et al. 2011, Hoot et al. 2012, Jiang et al. 2017 and references therein). Thus, the recognition of the newly outlined *Anemonastrum* will also allow continued generic recognition of *Hepatica*, a group very well distinguished morphologically, which was widely accepted as a separate genus in many standard floras and other publications (e.g., Juzepczuk 1937; Steyermark and Steyermark 1960; Duncan and Keener 1991; Tutin and Chater 1993; Tamura 1993, 1995; Czerepanov 1995; Fu and Robinson 2001; Uotila 2001; Luferov 2004; Malyshev 2012; Tzvelev 2012). At present, nomenclatural combinations for many species and several infraspecific and infrageneric entities in *Anemonastrum* already exist; they were validated mainly by Holub (1973) and later by some other authors (Löve and Löve “1975” (published 1976); Starodubtsev 1989, 1991; Raus 2011a, 2011b; Tzvelev 2012; and others). Several new nomenclatural combinations in *Anemonastrum* (mainly for North American taxa) have been recently validated by Mosyakin (2016). Additional nomenclatural transfers are now considered in parallel with continued taxonomic reassessment of *Anemone* sensu lato (Ziman et al. in prep.).

Christenhusz and Byng (in Christenhusz et al. 2018: 73) briefly discussed the recent molecular phylogenetic publications on Anemoneae and also advocated the recognition of several genera segregated from *Anemone* sensu lato. In particular, they recommended to recognize the following genera: *Anemone*, *Anemonidium*, *Eriocapitella* Nakai, *Knowltonia*, *Hepatica*, and *Pulsatilla* (Christenhusz et al. 2018: 73), and proposed new combinations for some species in *Anemonidium*, *Eriocapitella*, and *Knowltonia*. The principles of selection of species for these new combinations remain unclear to us because many other taxa of these groups were left untouched by these authors. Fortunately, Christenhusz et al. (2018: 1) included the following explanation (which is rather unusual, as for nomenclatural publications): “Inevitably we will have omitted some combinations, but this is not intentional. It is also possible that new combinations already existed but were not included in any of the standard databases cited above and hence we may have overlooked these. We apologize for these discrepancies and unintentional superfluous names, and we shall correct errors in future updates”.

Moreover, Christenhusz and Byng (in Christenhusz et al. 2018: 73) evidently did not notice that the generic name *Anemonastrum* (Holub 1973) is of priority over *Anemonidium* (Holub 1974) and, among other nomenclatural novelties, proposed the new combination *Anemonidium narcissiflorum* (L.) Christenh. & Byng for *Anemone narcissiflora* L., which is the type of Anemone sect. Omalocarpus DC., and thus also the type of the replacement name *Anemonastrum* (see Holub 1973: 158). Consequently, the name *Anemonastrum* should be used for the genus in that particular circumscription, as it has been already indicated by Mosyakin (2016).

Considering the various nomenclatural options and available phylogenetic and morphological evidence, we conclude that segregation of several genera from *Anemone* sensu lato is at least strongly preferable, if not inevitable. On the other hand, we believe that the generic over splitting of *Anemone* sensu lato in general and the *Anemonastrum* group in particular into numerous “narrow” genera, as proposed by Starodubtsev (1989, 1991, 1995) and accepted by some other authors (e.g., Czerepanov 1995; Malyshev 2012; Tzvelev 2012), should not be recommended, partly because some of the proposed generic segregates are in fact unnatural non-monophyletic assemblages of phylogenetically quite unrelated taxa. Most of recent taxonomic revisions of various groups of *Anemone* sensu lato or its infrageneric groups (Tamura 1993; Tutin and Chater 1993; Dutton et al. 1997; Wang et al. 2001; Luferov 2004; Ziman et al. 2004a, 2004b, 2004c, 2005, 2006a, 2006b, 2007, 2008; Ehrendorfer et al. 2009) usually applied a rather traditional generic concept, with recognition of *Hepatica*, *Pulsatilla*, and a resulting paraphyletic *Anemone*.

Here we propose new combinations for two species from the Southern Hemisphere, which clearly belong to *Anemonastrum* in its new circumscription and are interesting outliers from a biogeographic and conservation viewpoint.

## Taxonomic history of *Anemone tenuicaulis* and *A. antucensis* and their biogeographic links

**Figure 1.**
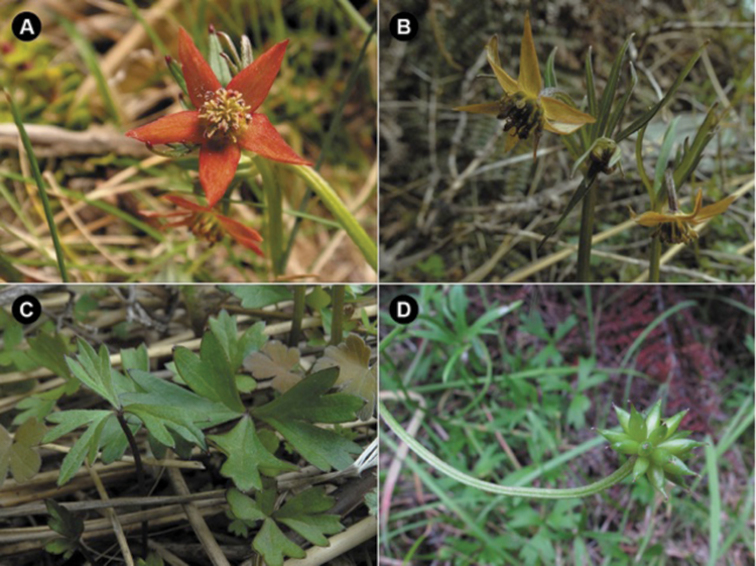
*Anemonastrum tenuicaule*. **A** Flowering plant. Hunter Mountains, Fiordland, South Island, New Zealand (photo: J. Bythell) **B** Flowering plant, Southland, South Island, New Zealand (photo: R. Hindmarsh-Walls) **C** Basal leaves, Southland, South Island, New Zealand (photo: R. Hindmarsh-Walls) **D** Fruiting plant, Minaret Burn, Otago, South Island, New Zealand (photo: J.W. Barkla)

**Figure 2.**
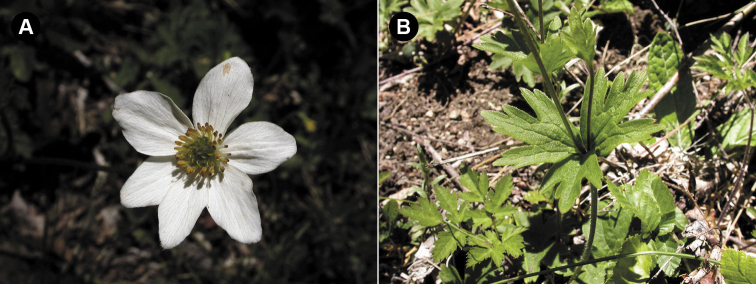
*Anemonastrum antucense*. **A** Flower, Parque Nacional Nahuelbuta, Chile, South America **B** Foliage – showing basal leaves, cauline leaves, and bracts, Parque Nacional Nahuelbuta, Chile, South America (photos: P. B. Pelser).

The species widely accepted until recently as *Anemone tenuicaulis* (Cheeseman) Parkin & Sledge was originally described from New Zealand by Cheeseman (1885) as a species of *Ranunculus* L., *R. tenuicaulis* Cheeseman. At the time of its recognition Cheeseman (1885) commented that his new species is a “very distinct and well-marked plant” (Fig. 1), and indeed it was considered an oddity in the New Zealand flora. The species was accepted in *Ranunculus* in New Zealand Flora treatments (e.g., Kirk 1899; Cheeseman 1906, 1925) until the 1930s, when Parkin and Sledge (1935) provided reliable morphological evidence for the placement of that taxon in *Anemone*. In that paper they also discussed its possible biogeographic links with the South American species *A. antucensis* Poepp. (Poeppig 1833) (Fig. 2) and the Tasmanian taxon *A. crassifolia* Hook. (Hooker 1840). Since 1935, the New Zealand species was commonly accepted as *Anemone tenuicaulis* (e.g., Allan 1961; Webb et al. 1988; de Lange 2004; de Lange et al. 2006; de Lange and Rolfe 2010; Schönberger et al. 2017) and its placement in *Anemone* was not challenged. However, Christenhusz and Byng (in Christenhusz et al. 2018: 73) recently transferred it to *Anemonidium*, as *A. tenuicaule* (Cheeseman) Christenh. & Byng, but in fact in their circumscription the genus should be called *Anemonastrum* (see comments above and our new combination below).

The species is a biologically sparse, naturally uncommon plant of mountain areas of the southern North and South Islands of New Zealand (Allan 1961; Webb et al. 1988; de Lange 2004). Its current conservation status is “At Risk – Naturally Uncommon” (de Lange et al. 2009; de Lange et al. 2013).

The geographical proximity of New Zealand *Anemone tenuicaulis* and Australian *A. crassifolia* has tempted many authors to hypothesize on their close relationships (Parkin and Sledge 1935; Hoot et al. 1994; Schuettpelz and Hoot 2000). That opinion was accepted in recent Australian floras. For example, Eichler and Jeanes (2007: 297) commented that the closest ally of *A. crassifolia* “appears to be the New Zealand *A. tenuicaulis* (Cheeseman) Parkin & Sledge, which is the only other Australasian *Anemone*. Its affinities are closer to South American species of Anemone sect. Rivularidium Jancz. than to Asian species” (also cited by Duretto 2009: 5).

Only reliable molecular phylogenetic evidence finally demonstrated the positions of the New Zealand and Tasmanian species in two distant clades (in fact, different genera, as accepted here) and the relatedness of *A. tenuicaulis* and *A. antucensis* (Ehrendorfer and Samuel 2000, 2001; Schuettpelz et al. 2002; Hoot et al. 2012). *Anemone crassifolia* was reported positioned in the clade of Anemone sect. Pulsatilloides DC. (sensu Hoot et al. 2012), its subclade consisting of several South American taxa, including those earlier placed in genera *Barneoudia* and *Oreithales*. Christenhusz and Byng (in Christenhusz et al. 2018: 75) recently transferred the Tasmanian species to *Knowltonia* as *K. crassifolia* (Hook.) Christenh. & Byng. This transfer is in line with the earlier suggestion by Mosyakin (2016), who proposed to recognize *Knowltonia* in an expanded circumscription and was preparing corresponding nomenclatural transfers (which are, of course, not needed now).

*Anemone tenuicaulis* has the base chromosome number *x* = 7 (2*n* = 28) (Hair 1963, Ehrendorfer 1995, Ziman et al. 2006), which is typical for all those members of *Anemonastrum* and *Hepatica*, for which chromosome numbers are known. In contrast, the base chromosome number *x* = 8 is reported for *A. crassifolia* (Schuettpelz et al. 2002), which indicates its position in another large clade of Anemoninae containing typical representatives of *Anemone* sensu stricto and some other subclades. Interestingly, Ziman et al. (2006) reported for *A. crassifolia* the chromosome numbers *x* = 7 (with reference to Huynh 1970) and *x* = 8 (referenced to Schuettpelz et al. 2002); however, the article by Huynh (1970) contains no data on chromosome numbers of that species. Thus, the indication of *x* = 7 for *A. crassifolia* was erroneous and probably caused by some misunderstanding.

Palynomorphological data also indicate that *Anemone tenuicaulis* and *A. crassifolia* are not related: spiroaperturate pollen grains of *A. crassifolia* are fundamentally different in their morphology from tricolpate pollen of *A. tenuicaulis* and *A. antucensis* (Huynh 1970; Moar 1993). Despite that fact and some other morphological differences, Huynh (1970: 93) rather paradoxically concluded that *A. tenuicaulis* “is probably more closely related to the Tasmanian *A. crassifolia*, in spite of a marked difference in their habit”.

Judging from the available morphological, taxonomic, biogeographic, and molecular phylogenetic data, *Anemonastrum* (in the circumscription accepted here) most probably initially diversified somewhere in East Asia and/or the Beringian region. From that hypothetical center of origin and early diversification, some representatives of the genus migrated westward to western and partly southern Asia (forming secondary centers of diversity, e.g. the Himalayas: see Ziman et al. 2001; 2007, Elliott 2016) and other regions of Eurasia (Ziman et al. 2005, 2006a), while another ancestral stock migrated eastward to North America. From North America some taxon (or taxa?) dispersed to the mountains of South America, and then from southern South America to New Zealand, possibly via Antarctica (see Meudt 2006, Winkworth et al. 2015). Cases of amphitropical disjunctions of North and South American plant taxa though uncommon are not unique (see an overview in Simpson et al. 2017 and references therein). It is also postulated that some groups of plants reached New Zealand from South America either by direct long-distance dispersal between those regions or via movement across Antarctica (see Raven 1973; Pole 1994; Macphail 1997; Winkworth et al. 1999; Wardle et al. 2001; Winkworth et al. 2002, 2005; Winkworth et al. 2015; Mosyakin et al. 2007; Meudt 2006; Sanmartín et al. 2007 and references therein). Alternatively, some genera may have been ‘shed’ from Antarctica into South America, New Zealand and Australia as conditions in Antarctica deteriorated and the land became fully ice-bound (Wardle et al. 2001). That said, the case for movement of biota along the Antarctic continent or outward dispersal from there though widely postulated, needs more critical assessments. With respect to New Zealand, this is especially so as the alpine region of that country was scarcely developed when Antarctica became fully ice-bound (Heenan and McGlone 2013).

As both *A. tenuicaulis* and *A. antucensis* have hooked or even spirally curved styles on tops of achenes, which are hardened in fruit, they are capable of being attached to animals (zoochorous dispersal, epizoochory). Thus, zoochory (most probably ornithochory, dispersal by birds – see Thorsen et al. 2009) may also have facilitated the migration of an ancestor of *A. antucensis* from North America to South America and subsequent migration of an ancestor of *A. tenuicaulis* from South America to New Zealand.

Schuettpelz and Hoot (2000) initially considered a possibility of the direct migration of an ancestral taxon of *A. tenuicaulis* from Asia to New Zealand. However, Ehrendorfer and Samuel (2000: 783), commented that the “suggestion of a direct dispersal from Asia to New Zealand (Schuettpelz and Hoot 2000) is not compatible with the much closer molecular affinity of *A. tenuicaulis* with the South American *A. antucensis* than with the Northern Hemisphere species pair *A. dichotoma* + *A. canadensis*”. Additional molecular data suggested that the South America – New Zealand disjunction in this case is better explained by a long-distance (or step-stone?) westward migration event (Schuettpelz et al. 2002; Hoot et al. 2012). It is not yet clear whether it was a direct dispersal from South America, or movement via intermediate stations in unglaciated parts of Antarctica sometime in the Tertiary.

The age estimates of the South America – New Zealand disjunction in the case of *Anemone* sensu lato remain controversial. Ehrendorfer and Samuel (2000: 783) mentioned that for the *A. antucensis*/*A. tenuicaulis* disjunction “one might speculate a late Miocene age” and that for pre-Pliocene migrations “the still more or less unglaciated Antarctic evidently has been an important link and transit area”. Considering the close relationships and probably quite recent time of divergence of *A. antucensis* and *A. tenuicaulis*, the hypothesis of migration of a founder species to New Zealand *via* yet unglaciated parts of Antarctica or through some other formerly existing hypothetical landmasses or land bridges (as initially hypothesized by Parkin and Sledge 1935) is possible but less probable than the preferred North America – South America – New Zealand long-distance dispersal. It is also worth noting that very similar phylogenetic and biogeographical patterns were revealed for representatives of another genus of Ranunculaceae, *Caltha* L. (see Schuettpelz and Hoot 2004), as well as for some genera from other families.

## Possible biogeographic links of two host-specific species of smut fungi parasitizing *Anemone antucensis* and *A. tenuicaulis*?

Additional indirect evidence of a phylogenetically isolated position of *Anemone antucensis* among other South American species of *Anemone* sensu lato is available from the fields of mycology and phytopathology. In particular, many of taxa of *Anemone* sensu lato are parasitized by *Urocystis anemones* (Pers.) G. Winter, a smut fungus widespread in the Holarctic (Denchev et al. 2000) but in South America known only on the Chilean *Anemone decapetala* Ard. (Piątek 2007, and references therein). However, *Urocystis antucensis* (Liro) M. Piątek seems to be an endemic species reported only on *A. antucensis* from Chile. Piątek (2007: 96) commented that since the time when *Tuburcinia antucensis* Liro (1922), the basionym of *Urocystis antucensis*, was described, it “has been completely forgotten and not reassessed by any smut taxonomist. Although I originally expected this species to represent one of the already known *Urocystis* species on various *Anemone* species described from elsewhere, I was surprised to find that it is a distinct and separate species”.

*Anemone tenuicaulis* is also parasitized by a host-specific smut fungus apparently endemic to New Zealand, *Urocystis novae-zelandiae* (G.Cunn.) G.Cunn. (Vánky and McKenzie 2002; Piątek 2007). Earlier records of *Urocystis anemones* on New Zealand’s species of *Ranunculus* are erroneous and in fact belong to another species of smut fungi, *Urocystis ranunculi* (Libert) Moesz (see McKenzie and Vánky 2001, 2002). *Urocystis novae-zelandiae* is listed in New Zealand as “Data Deficient” because it is known from so few collections (Hitchmough and Bull 2005). However, it has also been listed as “Vulnerable” by *The Global Fungal Red List Initiative* (Denchev et al. 2015) though on what basis is not clear, as its host plant is not similarly threatened but rather a naturally uncommon, biologically sparse species of mostly secure montane to alpine habitats in New Zealand (de Lange et al. 2013, as *Anemone tenuicaulis*). It is more likely that *Urocystis novae-zelandiae* is being overlooked rather than that it is truly threatened.

It would be interesting to check, using molecular and morphological approaches, if these two species of parasitic fungi, *U. antucensis* and *U. novae-zelandiae*, are related (or not?). If those two fungal species are proved to be indeed related, then their biogeographic patterns are identical to those of their hosts and probably resulted from the same long-distance dispersal event (or events?). If these species are not related, then a host-jumping event and parallel adaptation of parasites to related hosts most probably occurred. At present, ten smut genera are reported as endemic for Australasia, and that number of endemic genera in this group is exceptionally high as compared to all other continents, “which may point at fast evolving characters and/or may be caused by the regional history, including the long-term geographic isolation of Australasia” (Lutz et al. 2012: 143).

## Validation of new combinations

Acronyms of herbaria are given below following *Index Herbariorum* (Thiers 2018–onward).

### Anemonastrum antucense

Taxon classificationPlantaeRanunculalesRanunculaceae

(Poepp.) Mosyakin & de Lange comb. nov.

urn:lsid:ipni.org:names:60476483-2

≡ Anemone antucensis Poepp., *Fragm. Syn. Pl.*: 27. 1833. **Lectotype** (designated by Britton 1892: 229; designation confirmed and specified here) . CHILE. Bío Bío Province: Field label (*manu* Poeppig?): “No. 751. Anemone. A.”. Printed label: “(Pöppig Coll. pl. Chil. III) 150. Anemone antucensis Kz. | Syn. pl. Amer. austr. msc. | Diar. 751 | In Chil. austr. sylv. alpin. Andes de Antuco. | Decbr. lecta”. Curatorial label: “Herb. Mus. Paris | Amérique Méridionale. Poeppig. (1868 [the date of provenance?—S.M. & P.dL.], No. 34)” (P00585248!; **Isolectotypes**: G? fide Ziman *et al*. 2006: 2017, as “lectotype”, *non vidi*, HAL0077581!, BPI181305! fragments of leaves from a syntype, affected by Urocystis).

#### Notes.

Ziman et al. (2006: 217) provided the following type information on *Anemone antucensis*: ‘Type: Chile australes, silvis alpinis, Pico de Pilque”, 12.1832. Poeppig 751 (lectotype—G; isolectotype—P!)’. However, Britton (1892: 229) much earlier listed a specimen (syntype) “Poeppig 150” and noted that the “Type in the Paris Herbarium”. We were able to find information on only one syntype of *A. antucensis* deposited in P. Consequently, Britton’s type designation should be followed and the lectotype of *A. antucensis* is the specimen P00585248 cited above, while a specimen from G is thus considered an isolectotype.

### Anemonastrum tenuicaule

Taxon classificationPlantaeRanunculalesRanunculaceae

(Cheeseman) de Lange & Mosyakin comb. nov.

urn:lsid:ipni.org:names:60476484-2

≡ Anemonidium tenuicaule (Cheeseman) Christenh. & Byng in Christenhusz et al. (Eds) *The Global Flora* 4: 73. 2018.
≡ Anemone tenuicaulis (Cheeseman) Parkin & Sledge, *J. Linn. Soc., Bot.* 49: 647. 1935.
≡ Ranunculus tenuicaulis Cheeseman, *Trans. & Proc. New Zealand Inst.* 17: 235. 1885. **Lectotype** (designated by Allan 1961: 164; accepted by Burrows 1986: 15, and confirmed and specified here). NEW ZEALAND. South Island, Mountains above Arthur’s Pass, Canterbury Alps. Printed and handwritten label: “Herb. T.F. Cheeseman | Ranunculus sp. [“sp.” crossed out—S.M. & P.dL.] tenuicaulis n. sp. [new identification added in pencil—S.M. & P.dL.] | Locality:—South Island, N.Z. | Mts above Arthur’s Pass, Canterbury Alps, alt. | 4,500 ft. | Jany [January—S.M. & P.dL.] 1883 | Collector—T.F.C.” Small slip attached in the upper part of the sheet: “TYPE SELECTED. Dec. 1941. [signature of Lucy Cranwell]” (AK4232!; **isolectotypes**: “Herb. T.F. Cheeseman. **Com.** [communicated?] **9/83** [text in bold added in black ink, handwritten—S.M. & P.dL.] | Ranunculus n. sp.? | LOCALITY: —South Island, N.Z. | mountains above Arthur’s Pass, Canterbury, | alt. 4,500 ft. January 1883 | Collector—T.F.C. [T.F. Cheeseman—S.M. & P.dL.]”. Identification added directly on the sheet under the label: “Ranunculus tenuicaulis, Cheeseman” K000692121!, reported by Ziman *et al*. 2006: 217 as “lectotype”, E *s.n.* reported by Ziman *et al*. 2006: 217, *non vidi*).

#### Notes.

Cheeseman (1885) reported his new species (as *Ranunculus tenuicaulis*) from “Canterbury mountains above Arthur’s Pass, altitude 4,000–5,000 feet. *T.F.C.*” and all his collections from that locality should be considered syntypes. Ziman et al. (2006: 217) provided for *Anemone tenuicaulis* the following type information: “Type: NEW ZEALAND. South Island, Auckland, South Alps, Mountains above Arthur’s Pass, Canterbury, 4000–5000 ft. 1.1883. Lannary (lectotype—K!; isolectotype—E!)”. They, however, cited “Auckland” (printed on the label, indicating the location of Cheeseman’s herbarium) as part of the type locality information, misunderstood the handwritten word “January” for a collector name (“Lannary”), and erroneously listed the combination *Anemone tenuicaulis* as validated in “Nat. 1 (1932)”, the incomplete citation evidently corresponding to the article in *Nature* (Parkin and Sledge 1932) in which only preliminary information on the new generic placement of *Ranunculus tenuicaulis* was reported, but no new combination has been validated. When listing and designating types of *Ranunculus* names from New Zealand, Garnock-Jones (1990) only mentioned *Ranunculus tenuicaulis* among the taxa that are excluded from that genus but gave no type information.

The following type information was provided by Allan (1961: 164): “Type locality: “Mountains above Arthur’s Pass, alt. 4000–5000 feet.” Type: A, T. F. Cheeseman”, which constitutes effective lectotypification (Art. 7.10 of the ICN: McNeill et al. 2012). In this citation, the letter “A” indicates the Herbarium of Auckland Institute and Museum (AK). Burrows (1986) in his article also provided a table entitled “List of vascular plant taxa described originally from Arthur’s Pass National Park” and listed *Anemone tenuicaulis* (*Ranunculus tenuicaulis*), with proper references to the authors of the basionym and combination and their original publications. He reported (Burrows 1986: 15) the date and place of the original collection of Cheeseman (“Jan 1883 Mts above Arthur’s Pass”) and the location of the type specimen (“AUCK”, meaning “Auckland Institute & Museum [AK]”; see explanation in Burrows 1986: 17). Considering the lectotypification information provided above, the Kew specimen is not the lectotype of *Ranunculus tenuicaulis*, but an isolectotype.

There are several specimens of the species at AK collected by Cheeseman, e.g., AK4233, AK4234 (data and images available from the Auckland War Memorial Museum: http://www.aucklandmuseum.com), but only one collected in January 1883 near Arthur’s Pass and matching other data provided by Allan (1961) and Burrows (1986). Lucy M. Cranwell, who incorporated the Cheeseman collections (ca. 10 000 specimens) into AK, in December 1941 annotated the specimen AK4232 as the type (see above), but her type designation was not formally published. It is documented (Goulding 1974, 1975, 1976) that Cheeseman exchanged herbarium specimens with several European, American, and Australian herbaria and individual botanists; thus, additional isolectotypes or syntypes could be found in some other collections, in addition to the specimens known to be at K and E.

## Supplementary Material

XML Treatment for Anemonastrum antucense

XML Treatment for Anemonastrum tenuicaule
